# Molecularly Templated Buried Interfaces for Inverted Perovskite Solar Cells

**DOI:** 10.1002/anie.6932848

**Published:** 2026-08-07

**Authors:** Songyang Yuan, Quanrun Qiu, Huaiman Cao, Chengda Ge, Yiting Jiang, Gengyang Su, Lingyi Ke, Gengxin Du, Guangruixing Zou, Hui Liu, Guanhua Ren, Jing Wang, Nan Zhang, Yidan An, Qingduan Li, Ze Yu, Tao Jia, Yue‐Peng Cai, Shengjian Liu, Hin‐Lap Yip

**Affiliations:** ^1^ School of Chemistry Guangzhou Key Laboratory of Materials for Energy Conversion and Storage Key Laboratory of Electronic Chemicals For Integrated Circuit Packaging South China Normal University (SCNU) Guangzhou China; ^2^ Department of Materials Science and Engineering City University of Hong Kong Kowloon Hong Kong China; ^3^ State Key Laboratory of Fine Chemicals Frontiers Science Center For Smart Materials Oriented Chemical Engineering School of Chemical Engineering Dalian University of Technology Dalian China; ^4^ Guangdong Zenithnano New Material co., Ltd Guangzhou China; ^5^ Hong Kong Institute For Clean Energy (HKICE) City University of Hong Kong Kowloon Hong Kong China; ^6^ State Key Laboratory of Marine Environmental Health City University of Hong Kong Kowloon Hong Kong China; ^7^ School of Energy and Environment City University of Hong Kong Kowloon Hong Kong China

**Keywords:** buried interface engineering, charge extraction, inverted perovskite solar cells, molecular templating, self‐assembled monolayers

## Abstract

The buried interface between self‐assembled monolayers (SAMs) and perovskite absorbers critically governs charge extraction and stability in inverted perovskite solar cells, yet remains structurally mismatched and poorly controlled. Here, we report a buried‐interface engineering strategy inspired by non‑covalent molecular templating, enabled by complementary triphenylamine‐based molecular building blocks. A triphenylamine‐based ammonium salt, 2‐(4‐(diphenylamino)phenyl)ethanammonium iodide (TPANI), is introduced into the perovskite precursor, while a structurally matched triphenylamine‐based bisphosphonic acid SAM deposited on ITO serves as the hole‐selective layer. Non‐covalent interactions at the buried interface induce molecular templating and interfacial organization of TPANI, strengthening SAM/perovskite adhesion and reducing intergranular groove depth at the buried side of the perovskite film. The resulting monolithically integrated interface suppresses buried interfacial defects, improves energy‐level alignment, and facilitates hole extraction. Consequently, inverted devices achieve a power conversion efficiency of 26.58% with an exceptionally high fill factor of 86.72%, together with markedly enhanced operational stability. These results demonstrate that non‑covalent molecular templating provides an effective and general strategy for engineering buried interfaces in perovskite photovoltaics.

## Graphical abstract

We demonstrate a molecularly matched buried interface between SAM and perovskite, enhancing adhesion and passivating defects. This optimized interface achieves 26.58% power conversion efficiency and 86.72% fill factor with exceptional operational stability in both large‐area and wide‐bandgap devices, thereby increasing efficiency and prolonging operational longevity.

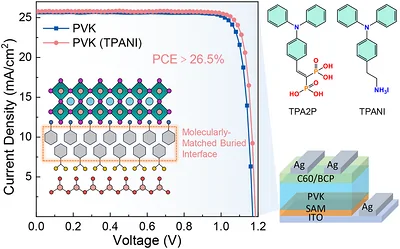

## 1 Introduction

Inverted perovskite solar cells (PSCs) based on self‐assembled monolayer (SAM) structures are a major research focus in the perovskite photovoltaic field, with certified power conversion efficiencies (PCEs) now exceeding 26%–27% [1, 2, 3, 4, 5, 6, 7]. As device efficiencies approach their practical limits, rational interfacial matching among functional layers has become a critical determinant of device performance. In particular, the buried interface between the SAM and the perovskite absorber plays a pivotal role in governing charge extraction, energy‐level alignment, and operational stability [8, 9, 10, 11], yet studies explicitly targeting this interface remain comparatively limited.

From a broader semiconductor perspective, the importance of such buried interfaces is well established. In inorganic semiconductor technologies, high‐performance devices rely on well‐defined heterojunctions, where strong interfacial coupling and conformal growth minimize electronic discontinuities and suppress interfacial recombination. While such epitaxial concepts cannot be directly translated to molecular–crystal junctions, they underscore the importance of interfacial adhesion, morphological continuity, and intimate electronic coupling for efficient carrier transport [12, 13, 14, 15, 16]. In this context, the SAM/perovskite interface in inverted PSCs is intrinsically heterogeneous, combining a molecularly thin organic layer with an ionic crystalline semiconductor [17, 18]. This fundamental mismatch often results in weak adhesion, morphological discontinuities, and a high density of buried interfacial defect states, ultimately limiting charge extraction and long‐term stability [7, 19].

Motivated by these interfacial challenges, researchers have employed a variety of molecular engineering strategies to improve the quality of the SAM/perovskite buried interface, primarily aiming to suppress interfacial defects and modulate energy‐level alignment [20, 21, 22, 23, 24, 25]. For example, Xu and coworkers designed a series of SAM molecules with modified linker groups to enhance molecular rigidity, systematically elucidating the influence of SAM rigidity on buried‐interface quality [26]. Yang and coworkers adopted a synergistic SAM strategy by incorporating an additive molecule, ABT (3,3′‐(4‐amino‐4H‐1,2,4‐triazole‐3,5‐diyl)dibenzoic acid), into Me‐4PACz, which effectively reduced SAM aggregation and yielded a denser and more uniform monolayer, thereby strengthening anchoring at the buried interface and alleviating interfacial charge recombination [27].

In parallel with SAM molecular design, incorporating functional additives into the perovskite precursor has also emerged as an effective route for regulating buried‐interface properties [28, 29, 30]. Zhu et al. introduced a novel n‐type small molecule, D4PA ((7H,7′H‐[5,5′‐bidibenzo[c,g]carbazole]‐7,7′‐diylbis(butane‐4,1‐diyl)) bis(phosphonic acid)), into the perovskite precursor. The intramolecular C─C bond coupling in D4PA enabled homogeneous and strong adhesion to both the perovskite and the substrate, resulting in improved defect passivation and interfacial charge transport, as well as outstanding operational stability, with over 97.2% of the initial efficiency retained after 2500 h of continuous operation at the maximum power point [7]. Similarly, Zang and coworkers incorporated a robust amidino‐based ligand, morpholine‐4‐carboximidamide hydrochloride (M4CH), into the perovskite precursor to promote (001)‐oriented film growth and induce a favorable interfacial dipole, thereby significantly enhancing carrier extraction [28].

Despite these notable advances, the overall effectiveness of buried‐interface modification strategies remains to be fully established. While passivating defect states at the buried perovskite interface via functional groups on SAM terminal moieties is widely adopted, achieving precise spatial correspondence between passivating groups and defect sites at the molecular level remains challenging [31, 32, 33]. Mixed‐SAM approaches provide greater flexibility for defect passivation, yet the compatibility and cooperative behavior of different molecular species within a single SAM layer require further investigation. More fundamentally, the intrinsic dissimilarity between the SAM and the perovskite—essentially a two‐phase molecular–crystal interface—inevitably affects energy‐level alignment and charge transport. As a result, interfacial matching between SAMs and perovskite absorbers warrants deeper and more systematic study [34].

Beyond the buried interface alone, simultaneous suppression of defect states at grain boundaries and buried interfaces is essential for obtaining high‐quality perovskite films with minimal non‐radiative recombination and efficient carrier transport. Molecular surface passivation is a widely used strategy to mitigate electronic defects arising from abrupt lattice termination; however, in halide perovskites such approaches can introduce complex effects, including unintended perturbation of crystallization behavior [35, 36]. Consequently, an ideal buried‐interface strategy should enhance interfacial coupling and morphological continuity without disrupting the bulk perovskite lattice or introducing additional structural complexity.

To address these requirements, herein we report a non‑covalent molecular‐templating buried‐interface engineering strategy enabled by complementary molecular design. An ammonium salt, TPANI (2‐(4‐(diphenylamino)phenyl)ethanammonium iodide), is incorporated into the perovskite precursor solution, while a structurally matched triphenylamine‐based bisphosphonic acid SAM, TPA2P ((2‐(4‐(diphenylamino)phenyl)ethene‐1,1‐diyl)bis(phosphonic acid)), previously demonstrated to function as an efficient hole‐selective layer [37], is employed as the interfacial contact. This deliberate molecular pairing establishes the foundation for interfacial matching during film formation. During perovskite crystallization, TPANI preferentially migrates to grain boundaries and the buried interface, where complementary non‐covalent interactions with the SAM enhance interfacial adhesion and promote conformal perovskite growth. This molecular templating process reduces intergranular grain‐boundary groove depth and yields a mechanically robust, well‐coupled SAM/perovskite junction that mimics key functional aspects of high‐quality inorganic heterojunctions.

As a direct consequence of this optimized interfacial matching, the buried interface effectively suppresses interfacial defect states, improves energy‐level alignment, and facilitates efficient hole extraction. Inverted PSCs fabricated using this strategy achieve a champion PCE of 26.58% with an exceptionally high fill factor of 86.72%. Unencapsulated devices retain 97.2% of their initial efficiency after 1000 h of continuous operation under 1.5G illumination in a nitrogen atmosphere. This work demonstrates that translating heterojunction design principles into molecular‐level interfacial templating provides a robust and general framework for engineering buried interfaces in next‐generation inverted PSCs.

## 2 Results and Discussion

### 2.1 Heterojunction Interface Design for Monolithic Integration

The synthetic route for TPANI could be found in our recent publication [38]. The molecular structures of TPANI and TPA2P are shown in Figure 1a. Based on our previous study, TPANI can combine with PbI_2_ forming an *n* = 1 Ruddlesden–Popper (RP) type 2D perovskite structure, (TPA)_2_PbI_4_. In this structure, the bilayer spacer cations (TPAN^+^) are tightly connected via head‐to‐head π–π stacking between the TPA terminal groups (Figure S1), primarily due to the strong dipole effect of TPAN^+^ ions, which promotes highly oriented alignment on the surface of the perovskite inorganic octahedral layers. Structurally, both TPA2P (used as the hole transport layer) and TPANI feature a triphenylamine (TPA) moiety. We therefore propose that the TPA terminal of TPA2P can interact with the TPA group of TPANI, forming a bilayer structure at the SAM/perovskite interface analogous to the spacer cation bilayer in (TPA)_2_PbI_4_ 2D perovskite. The key distinction is that this bilayer is formed with one layer originating from the SAM (TPA2P) and the other from TPANI, which is tightly bound to the perovskite inorganic layer, thereby monolithically integrating the SAM and perovskite layers via a molecularly coupled connection (Figure 1b,c). Such an interfacial design is expected to offer several advantages: (i) a highly effective defect‐passivating buried interface, (ii) efficient charge transport facilitated by π–π stacking between TPANI and TPA2P, and (iii) enhanced adhesion between the SAM and perovskite layers, thereby improving mechanical robustness and interfacial stability.

**FIGURE 1:**
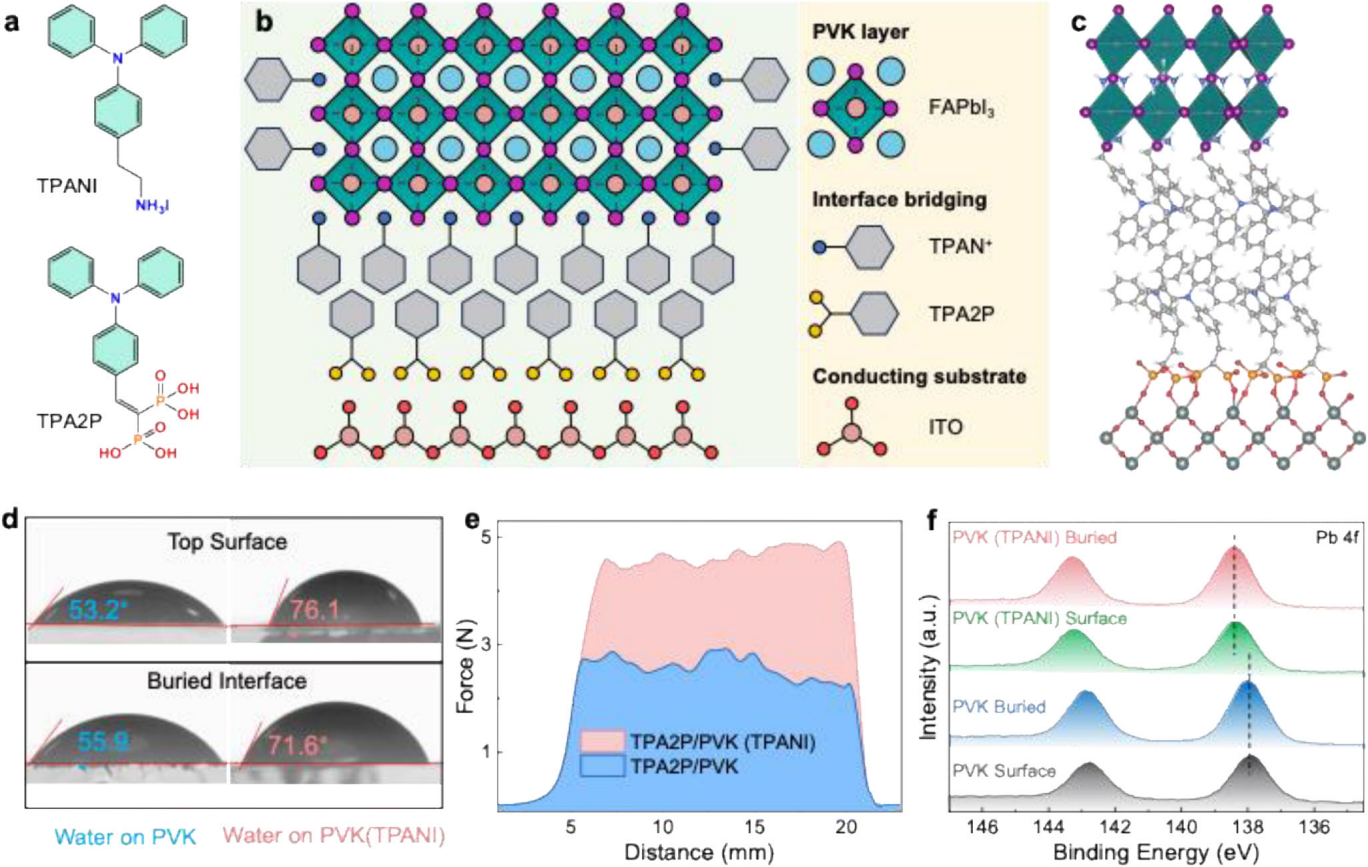
(a) The molecule structures of TPANI and TPA2P. (b) Schematic showing the monolithic integration by which TPAN^+^ connects the SAM and perovskite layers through a quasi RP type perovskite interface. (c) Interface structure diagram between the SAM and perovskite layers. (d) The contact angle of water droplet on the surface (the first line) of PVK and PVK (TPANI) films and water droplet on the buried interface (the second line) of PVK and PVK (TPANI) films. The perovskite films were carefully detached using glass sheet with ultraviolet curing glue, exposing the side of the perovskite layer that was in contact with the ITO/SAM substrate. (e) Tensile testing by peeling off perovskite film (w/wo TPANI additive) from ITO/TPA2P substrates. (f) XPS spectra of Pb 4f core level of perovskite films w/wo TPANI additive.

### 2.2 Morphological and Structural Evolution Upon TPANI Doping

To elucidate how TPANI influences perovskite crystallization and film morphology, we conducted systematic XRD and SEM analyses on perovskite films prepared with varying TPANI concentrations. At low doping levels (≤ 1 mg mL^−^^1^), the XRD patterns show only the characteristic reflections of the 3D perovskite phase, with no discernible peaks associated with low‐dimensional or quasi‐2D perovskite structures (Figure S2). As the TPANI concentration increases, additional reflections gradually appear at 7.1° and 10.5°, corresponding to the (002) and (003) planes of the layered perovskite (TPA)_2_PbI_4_, while the main 3D perovskite peaks remain essentially unchanged (Figure S3). These results suggest that at higher doping levels, TPANI can promote partial formation of a quasi‐2D perovskite–like structure through local structural reorganization during crystallization.

Among all tested compositions, the film doped with 0.75 mg mL^−^^1^ TPANI exhibits the strongest and sharpest 3D perovskite diffraction peaks, reflecting improved crystallinity. This optimized condition also yields the highest device performance (discussed later). Therefore, subsequent analyses focus on the 0.75 mg mL^−^^1^ TPANI‐doped film in comparison with the pristine perovskite. Although no clear 2D diffraction peaks are detected at this concentration, water contact angle measurements show a marked increase to over 70° for both the top and buried interfaces (Figure 1d), suggesting the formation of a hydrophobic organic layer likely composed of TPANI‐derived species at these interfaces. This observation implies that TPANI molecules preferentially segregate toward film surfaces and interfaces even at low doping levels, consistent with their limited incorporation into the perovskite lattice.

SEM images further reveal that excessive TPANI doping causes substantial morphological changes and disrupts surface uniformity (Figure S4), correlating with the reduction in device performance. Overall, moderate TPANI doping (∼ 0.75 mg mL^−^^1^) enhances crystallinity and induces a thin, hydrophobic interfacial layer beneficial for interfacial stability, whereas over‐doping deteriorates film morphology and electronic quality.

### 2.3 Interfacial Coupling and Chemical Interaction Mechanism

To probe the mechanical integrity of the buried interface, we measured the adhesion strength between the TPA2P layer and perovskite films with or without TPANI doping (Figure 1e). The onset force required to initiate delamination for the TPANI‐doped perovskite was approximately 4.7 N, compared to ∼2.8 N for the undoped control. The corresponding interfacial adhesion energies, derived from force–displacement integration, were 323.3 and 184.7 J m^−^^2^ for the doped and control films, respectively.

To assess molecular specificity, a control SAM based on 4PADCB ((4‐(7H‐dibenzo[c,g]carbazol‐7‐yl)butyl)phosphonic acid) was also examined. The TPANI‐doped perovskite film on 4PADCB exhibited a lower initial detachment force and adhesion energy (3.1 N, 214.8 J m^−^^2^) than that on TPA2P (Figure S5), highlighting the unique ability of TPA2P to form stronger interfacial coupling. This enhancement is attributed to favorable molecular interactions between the complementary TPA moieties of TPA2P and TPANI. Such π–π stacking and weak electronic coupling at the interface promote molecular coherence and mechanical robustness. Consistent adhesion results across five independent samples further confirm the reproducibility of this trend (Figures S6 and S7).

To further clarify the chemical nature of TPANI–perovskite interactions, x‐ray photoelectron spectroscopy (XPS) was conducted on both the top and buried interfaces. The Pb 4f core‐level peaks of TPANI‐doped films shifted to higher binding energy relative to the pristine perovskite (Figures 1f and S8). This shift reflects a decrease in electron density around Pb^2^^+^, consistent with partial passivation of undercoordinated Pb sites by the positively charged ammonium termini of TPANI. These ammonium groups can electrostatically interact with surface halides or [PbI_6_]^4^^−^ octahedra, mitigating surface defects and stabilizing the interfacial electronic environment. To further verify the depth‐dependent distribution of TPANI in perovskite film, we performed XPS depth‐profile analysis on the buried interface (Figure S9). The results show that the N 1s peak intensity from the TPA moiety significantly decreases with increasing depth. The NH_3_^+^ signal at the buried interface originates from the primary ammonium cations of TPANI. The weaker NH_3_^+^ signal compared to the N signal from the TPA moiety may be related to the orientational distribution of TPANI at the buried interface: the TPA groups are more exposed to the surface, while the NH_3_^+^ groups are located inward to bind with the perovskite layer. On the perovskite top surface (Figure S9b), the NH_3_^+^ signal is significantly weaker than that at the buried interface, further confirming that TPANI preferentially enriches at the buried interface during perovskite crystallization. The combined mechanical and spectroscopic results support a scenario where TPANI molecules preferentially accumulate at the buried interfaces, forming a passivating hydrophobic organic layer rather than substituting into the perovskite lattice at the optimal doping conditions.

Complementary DFT modeling further substantiates this interpretation. The calculated binding energy between TPAN^+^ and the perovskite surface (–2.83 eV) is markedly higher than that between TPA2P and perovskite (–0.96 eV), which corresponds to the interaction at the pristine perovskite/TPA2P on ITO interface (Supporting Information Note 1 and Figures S10 and S11). This result indicates that TPANI is thermodynamically more favorable to anchor at and stabilize the buried interface. Moreover, differential charge density analysis reveals pronounced electron transfer from the perovskite layer to the TPAN^+^ cations (Figure S12a), where the cyan isosurface region represents electron depletion and the yellow isosurface region represents electron accumulation, consistent with the Pb 4f binding energy upshift observed in XPS. Such charge redistribution lowers the density of undercoordinated Pb‐related trap states and enhances interfacial electronic coupling, collectively contributing to improved interfacial passivation and stability.

Together, the XPS and DFT results establish a coherent interfacial model in which TPANI forms robust electrostatic and van der Waals interactions with the perovskite surface. The complementary TPA moieties of TPA2P and TPANI further strengthen this coupling, leading to the formation of a chemically and mechanically stable heterointerface that underpins the improved device performance observed at optimal TPANI doping levels.

### 2.4 Morphology Optimization and Molecularly Complementary Interface Formation

The introduction of TPANI profoundly influences film formation dynamics and wettability at the buried interface. Contact angle measurements reveal that the perovskite precursor solution containing TPANI exhibits a smaller contact angle on the ITO/TPA2P substrate than the undoped solution (Figure 2a). This enhanced wetting behavior arises from the amphiphilic and bridging nature of TPAN^+^: while its TPA core interacts non‐covalently with the TPA2P monolayer, the ammonium terminus coordinates with perovskite precursors in solution. Consequently, TPAN^+^ mediates stronger interfacial affinity between the precursor and the TPA2P‐modified substrate, enabling more uniform film spreading—a key prerequisite for well‐crystallized perovskite layers.

**FIGURE 2:**
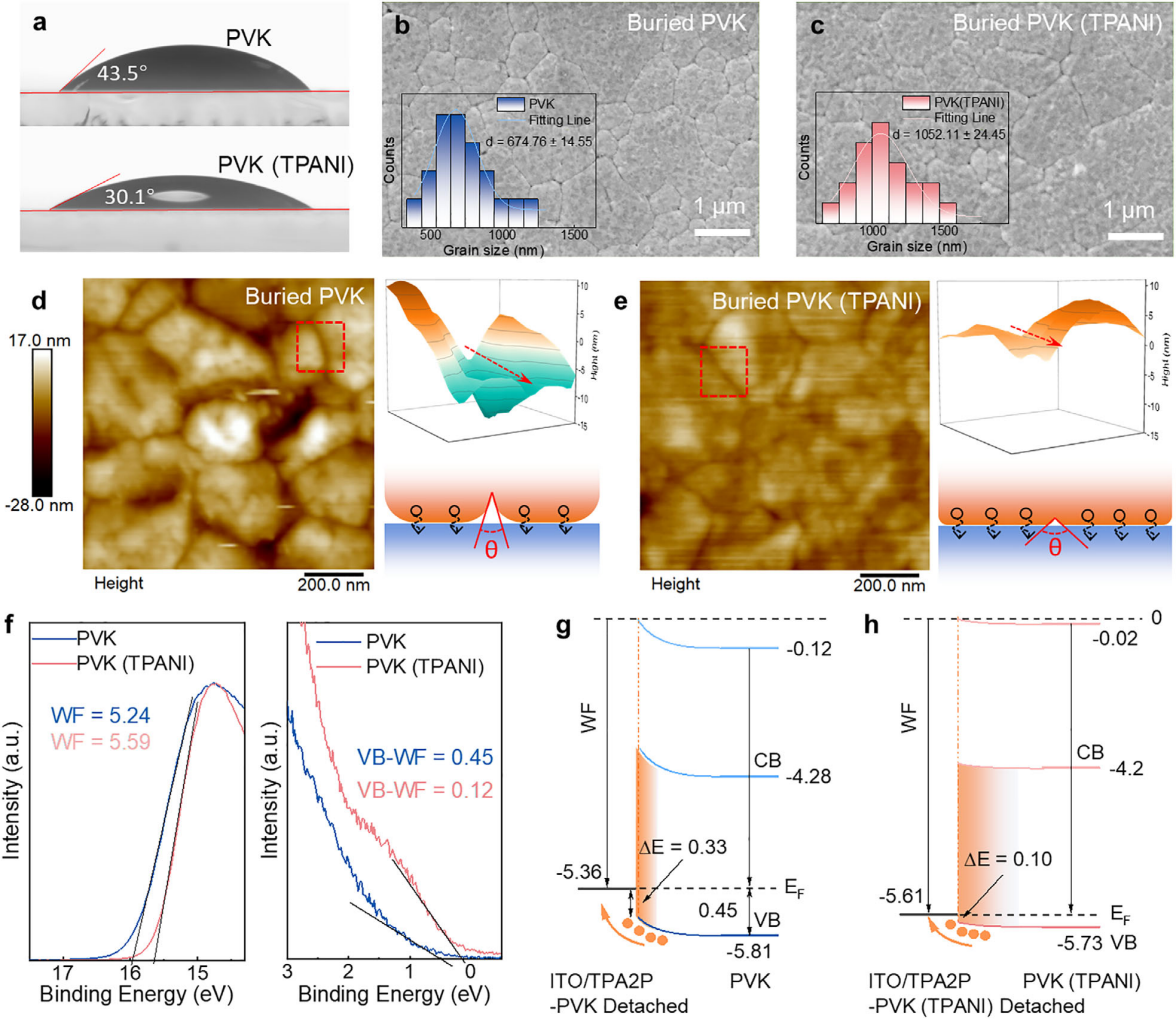
(a) The contact angle of perovskite precursor solution droplet (w/wo TPANI additive) on ITO/TPA2P substrates. (b,c) SEM images of the buried perovskite films (w/wo TPANI additive) deposited on ITO/TPA2P substrate. The inset figure showing the grain size statistical distributions of the corresponding perovskite films. (d,e) The AFM images of buried PVK interface w/wo TPANI additive. The 3D height surface maps corresponding to the positions of the red dashed boxes in the AFM images. The direction of the red arrows in the figures corresponds to the grain boundary direction. (d,e) Share the same scale bar. (f) UPS spectra of perovskite films w/wo TPANI additive. The plots on the left and right show the secondary electron cut‐off and valence band regions, respectively. (g,h) Energy‐level diagrams for (g) ITO/TPA2P/PVK and (h) ITO/TPA2P/PVK (TPANI). WF, work function; a.u., arbitrary units. VB, valence band; E_F_, Fermi level; CB, conduction band.

To probe the buried interface morphology, perovskite films were mechanically detached using UV‐curing glue to expose the surface initially in contact with the ITO/SAM substrate for SEM and AFM analyses (Figures 2b,c and S13). The TPANI‐modified film exhibits a smoother buried surface (Rq  =  3.9 nm) and larger grain size (≈1050 nm) than the undoped film (Rq =  5.8 nm; ≈ 675 nm). AFM height profiles (Figure 2d,e) show that TPANI flattens grain boundary channels—the channel depth decreases from 6.7 to 0.9 nm, while the boundary angle (θ) increases from ∼40° to >120°. These changes indicate that TPANI accumulates at grain boundaries during crystallization, where molecular complementarity with TPA2P termini reduces surface energy and promotes seamless grain coalescence. The resulting smoother, denser interface minimizes voids and interfacial roughness, ensuring better perovskite/SAM contact. This flattening suppresses local field concentration and mechanical stress, thereby mitigating ion migration and interfacial degradation, as supported by statistical AFM data across multiple regions (Figure S14).

These findings are consistent with the adhesion and XPS analyses, confirming that TPANI promotes the formation of a molecularly well‐organized, complementary interface at the boundary between the perovskite layer and the TPA2P monolayer. The synergistic interactions between the TPA moieties of TPANI and TPA2P, combined with electrostatic coordination between the ammonium group of TPANI and surface lead halide species, contribute to a tightly bound and defect‐suppressing interfacial arrangement. This molecular complementarity minimizes interfacial defects and facilitates efficient charge transfer across the TPA2P/perovskite (TPANI) junction. Furthermore, SEM and AFM measurements reveal a reduced surface roughness for the TPANI‐doped perovskite film (Figure S15), consistent with enhanced microstructural uniformity observed at the optimal doping concentration (0.75 mg mL^−^^1^; Figure S4).

### 2.5 Band Energy Alignment and Electronic Coupling at the Buried Interface

Ultraviolet photoelectron spectroscopy (UPS) was employed to elucidate how TPANI incorporation modifies the electronic structure at the buried interface. To access the interfacial region, the perovskite films were first mechanically detached from the ITO/TPA2P substrate, exposing the side originally in contact with the substrate for UPS measurements. As shown in Figure S16, the work functions (WFs) increase systematically from 4.81 eV for bare ITO to 5.21 eV for ITO/TPA2P, and further to 5.36 eV for ITO/TPA2P/PVK and 5.61 eV for ITO/TPA2P/PVK (TPANI) after film detachment. The progressive increase in WF indicates that TPANI incorporation induces a deeper Fermi level and a stronger interfacial dipole at the perovskite/TPA2P junction. These changes originate from electronic coupling between TPANI (and its PbI_2_‐chelated complexes) and the underlying TPA2P monolayer, rather than from the perovskite surface itself. Thus, the UPS results directly reflect the intrinsic band alignment and interfacial energy landscape of the buried TPA2P/perovskite (TPANI) interface.

Based on the Schottky contact model, the perovskite band at the ITO/TPA2P/PVK interface bends upward, exhibiting a substantial energy offset (ΔE ≈ 0.33 eV) (Figure 2f,g). While this band bending aids electron blocking and promotes hole extraction, an excessive ΔE reduces hole‐transfer efficiency due to diminished interfacial carrier concentration and thermionic losses, ultimately limiting open‐circuit voltage (*V_OC_*). In contrast, at the ITO/TPA2P/PVK (TPANI) interface (Figure 2h), the upward band bending persists but the hole‐injection barrier is significantly reduced (ΔE_h_ ≈ 0.02 eV) as a result of a deeper Fermi level and improved energetic matching. This optimized alignment effectively enhances hole extraction and interfacial charge transport. We attribute the improved energy‐level alignment primarily to electronic coupling between TPANI and TPA2P. Specifically, differential charge density analysis (Figure S12) reveals pronounced electron redistribution from the perovskite toward TPANI, and the strong adhesion enhancement is observed only for the complementary TPA2P/TPANI pair (Figure S6). These results indicate that interfacial π–π stacking generates a dipole, which accounts for most of the increase in WF and the reduction in band bending.

### 2.6 Defect Passivation and Photoelectric Performance of Perovskite Films

To elucidate the effect of TPAN^+^ on surface defect states, we calculated the electronic density of states (DOS) near the band edges before and after TPAN^+^ adsorption on the perovskite surface (Figures 3a and S17). In the pristine film, the valence band maximum (VBM) and conduction band minimum (CBM) originate mainly from C‐2p and I‐5p orbitals, respectively. Upon TPAN^+^ adsorption, the CBM shifts toward higher energy, indicating that TPAN^+^ functions as an electron donor. This electron‐donating behavior effectively passivates the dangling bonds related to I‐5p orbitals, producing an n‐type doping effect that mitigates surface electron traps. Charge density distributions at the VBM and CBM further confirm intense charge transfer between TPAN^+^ and perovskite surface (Figure 3b,c).

**FIGURE 3:**
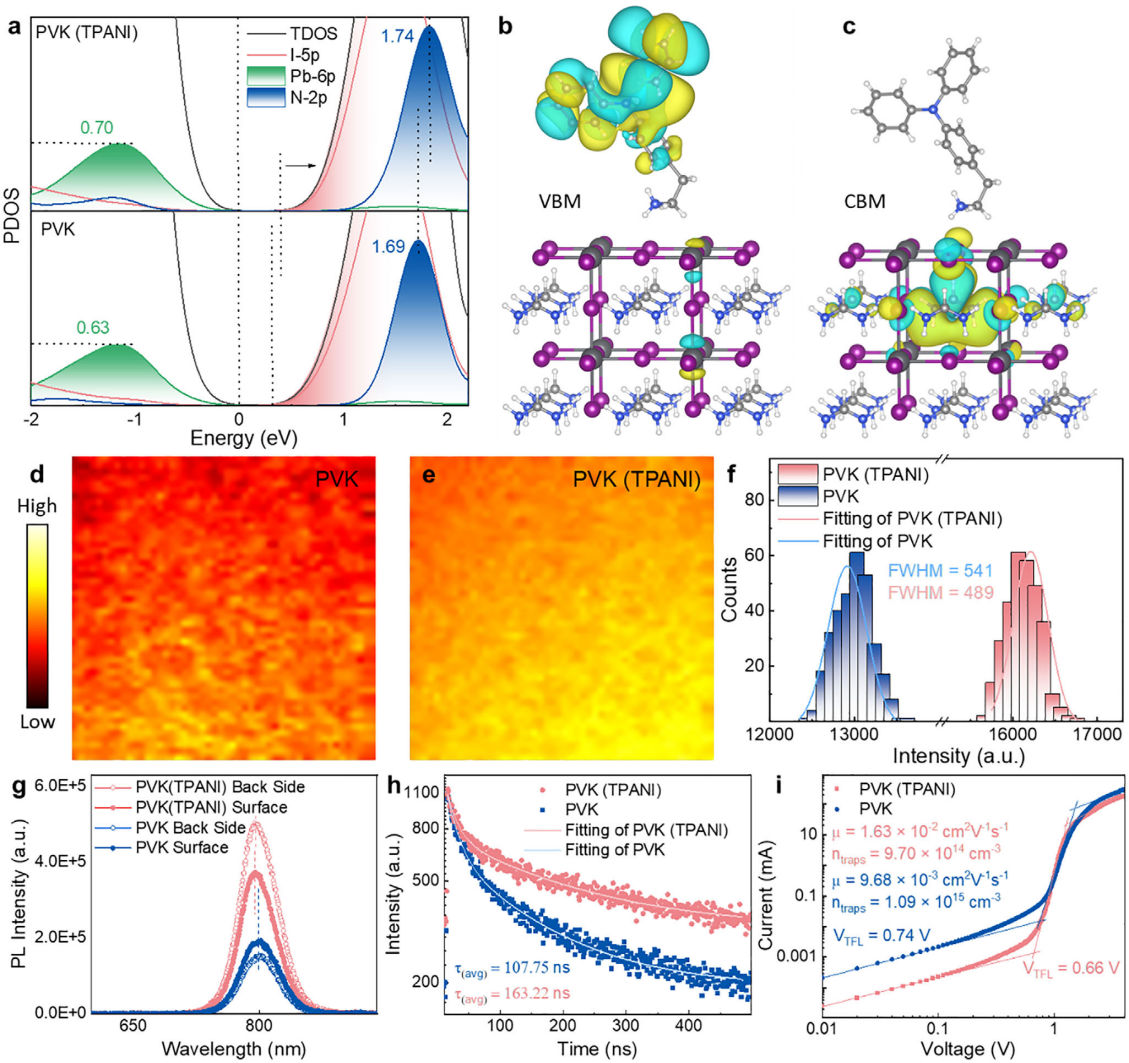
(a) The edge states of TDOS and PDOS distribution for PVK substrate and PVK substrate adsorbing one TPAN^+^ ion. (b) VBM and (c) CBM of PVK substrate adsorbing one TPAN^+^ ion. (d,e) PL mapping of the (d) perovskite films and (e) perovskite films with TPANI additive. (f) The PL intensity distribution extracted form (d,e). (g) PL and (h) TRPL spectra of perovskite films w/wo TPANI additive. (i) Dark *J‐V* curves for the hole‐only devices with an ITO/TPA2P/perovskite (w/wo TPANI additive)/PTAA/Ag structure.

Notably, the Pb‐6p orbital retains its energetic position but exhibits an increased DOS intensity (from 0.63 to 0.70), likely because the ─NH_3_^+^ group forms hydrogen bonds (N─H⋯I) with the perovskite surface. This interaction stabilizes the [PbI_6_]^4^^−^ octahedra and enhances orbital delocalization. Concurrently, the N‐2p orbital DOS slightly increases (1.69 → 1.74) and shifts toward higher energy. We attribute this to the formation of a positively charged dipole layer induced by the protonated amine group, which generates downward surface band bending. This electrostatic modification effectively pushes residual defect states away from the band edges, reducing non‐radiative recombination channels—an improvement conducive to higher *V_OC_*.

The impact of this defect suppression is experimentally corroborated by photoluminescence (PL) mapping at the buried interface over a 50 × 50 µm^2^ area (Figures 3d–f and S18). TPANI‐modified films exhibit brighter, spatially uniform PL emission, reflecting reduced trap‐mediated recombination. Moreover, the steady‐state PL peak shows a slight blue shift (Figure 3g), consistent with reduced defect‐induced sub‐bandgap emission. Time‐resolved photoluminescence (TRPL) measurements further confirm this improvement: the TPANI‐modified film exhibits an extended carrier lifetime (τ_2_ = 163.22 ns), nearly 1.5× longer than that of the control (τ_2_ = 107.55 ns;  Supporting Information Note 2 and Table S1). This prolonged lifetime evidences suppressed interfacial non‐radiative recombination, in agreement with the DOS and PL analyses.

To quantitatively evaluate interfacial recombination losses, we measured photoluminescence quantum yield (PLQY) and derived the quasi‐Fermi level splitting (QFLS), which reflects potential *V_OC_* losses (Supporting Information Note 3, Figure S19, and Table S2). The TPANI‐modified film achieves a higher PLQY (8.2%) than the control (3.8%), corresponding to a larger QFLS (1.198 eV) and a smaller *V_OC_* loss (64.7 vs. 84.5 mV). These observations collectively demonstrate that TPANI reduces non‐radiative recombination and enhances radiative efficiency at the buried interface. To further decouple whether the enhanced performance arises primarily from reduced bulk defects (due to improved crystallization) or from molecular templating at the buried interface, we performed additional QFLS measurements for perovskite films deposited on glass (bulk‐dominated reference) and on ITO/TPA2P, with and without TPANI. The introduction of TPANI yields a total QFLS increase of ∼19 mV on ITO/TPA2P (Table S2), whereas the bulk‐only contribution observed on glass is only ∼5 mV. Notably, the TPANI‐induced QFLS enhancement is therefore substantially larger on ITO/TPA2P than on glass, consistent with interface‐enabled recombination suppression that requires the complementary SAM. Collectively, these results indicate that buried‐interface molecular templating is the dominant contributor to the performance gain, rather than improved bulk crystallinity alone.

Finally, space‐charge‐limited current (SCLC) measurements were performed to assess trap densities and charge transport characteristics (Supporting Information Note 4 and Figure 3i). The TPANI‐modified hole‐only device shows a lower trap density (9.7 × 10^14^ vs. 1.09 × 10^1^ ^5^ cm^−^^3^) and nearly double the hole mobility (1.63 × 10^−^^2^ vs. 9.68 × 10^−^^3^ cm^2^ V^−^  s^−^^1^) compared to the control. Together, these improvements in passivation, defect suppression, and charge transport underscore the pivotal role of TPANI in stabilizing the perovskite lattice, promoting interfacial electronic order, and enhancing the overall optoelectronic performance.

### 2.7 Device Performance and Interfacial Impact on Photovoltaic Behavior

An inverted device architecture of ITO/TPA2P/perovskite (with or without TPANI)/C_60_/BCP/Ag was constructed to evaluate the photovoltaic performance of TPANI as a precursor additive for enhancing and unifying the buried interface between the SAM and perovskite layers (Figure 4a). For the normal bandgap devices with a composition of Cs_0_._05_FA_0_._95_PbI_3_, the benchmark control device (without TPANI) achieved a PCE of 25.65%, with a *V_OC_* of 1.170 V, a short‐circuit current density (*J_SC_*) of 25.65 mA cm^−^^2^, and a fill factor (*FF*) of 85.36% (Figures 4b, S20, S21, and Table S3). Incorporation of TPANI markedly improved performance, yielding a PCE of 26.58% with a stabilized power output (SPO) maintained between 26.49% and 26.16% after 400 s (compared with a decline from 25.08% to 24.19% for the control; Figure S22). The optimized TPANI‐based device exhibited a *V_OC_* of 1.186 V, *J_SC_* of 25.73 mA cm^−^^2^, and *FF* of 86.72%. The integrated current density (24.71 mA cm^−^^2^) from the external quantum efficiency (EQE) spectra (Figure 4c) closely matched the J–V data, with a deviation of less than 4%. The optical bandgap of derived from the first derivative of the EQE spectrum was 1.53 eV (Figure S23). The overall improvement is mainly attributed to enhanced interfacial passivation and energy‐level alignment enabled by TPANI, which together reduce *V_OC_* loss and facilitate charge extraction. TPANI also promotes improved crystallinity and reduces interfacial defect density, thereby contributing to the simultaneous enhancement of *J_SC_* and *FF*. We also analyzed the effect of the interfacial molecular templating on the hysteresis behavior of the devices (Figure S24). The Hysteresis Index (HI) is generally defined as [31]: HI = (PCE_Reverse_—PCE_Forward_)/PCE_Reverse_ × 100%. Using this definition, the HI decreases from 4.52% (control) to 2.33% (TPANI‐based device), indicating that TPANI–interface interactions suppress interfacial defects and bias‐induced ionic/electronic accumulation, thereby reducing interfacial recombination and ion migration, which are key factors for improved operational stability.

**FIGURE 4:**
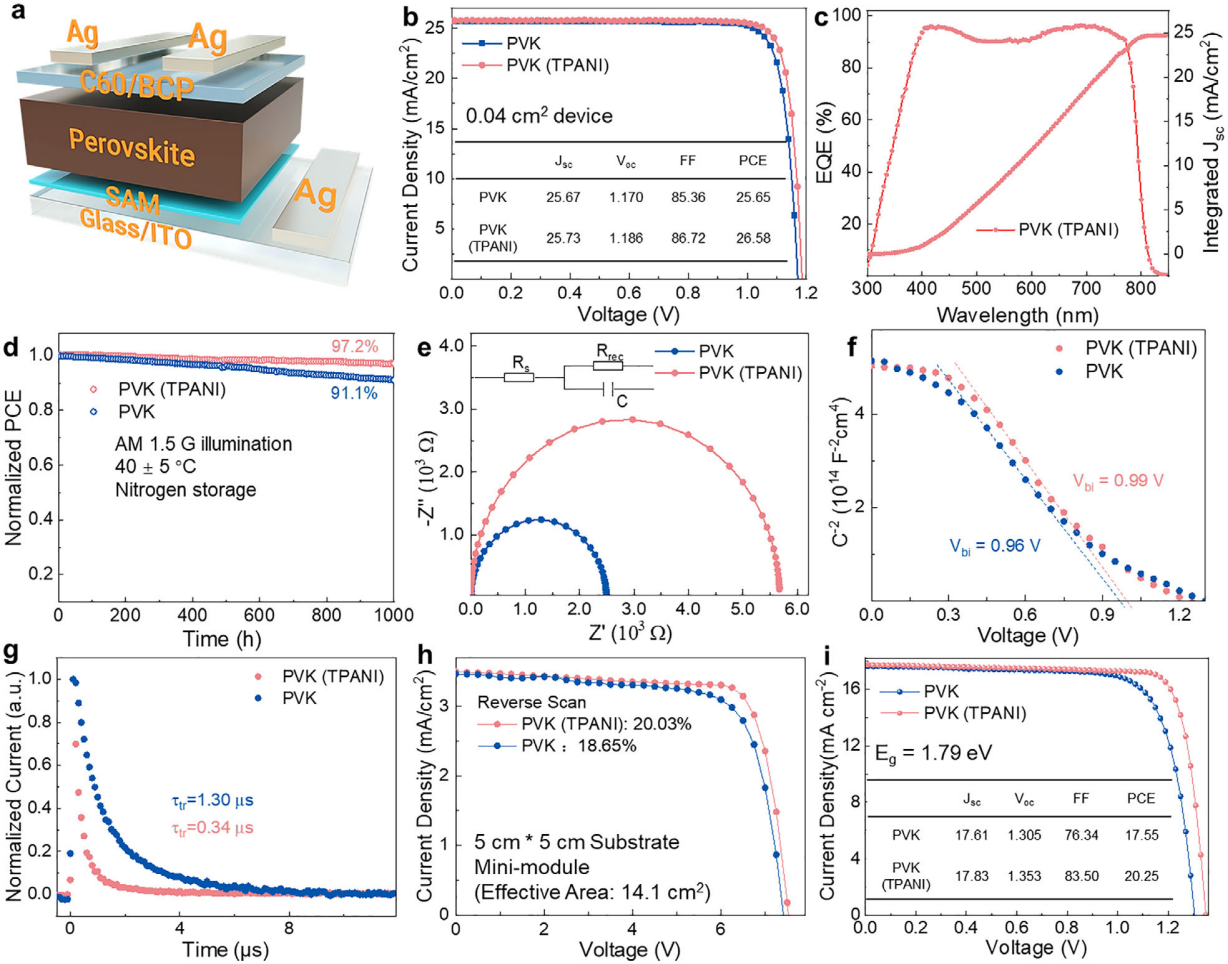
(a) Device structure of PSC. (b) Optimized *J‐V* curves of the perovskite (w/wo TPANI additive)‐based devices at reverse scan. (c) EQE spectra and integrated current densities of PVK (TPANI)‐based devices. (d) MPP tracking under N_2_ and simulated 1 sun AM 1.5 G illumination for uncooled devices (reaching an operating temperature of 40°C ± 5°C). (e) Nyquist plots, (f) Mott–Schottky plots and (g) TPC decay of the perovskite (w/wo TPANI additive)‐based devices. (h) *J‐V* curves of the perovskite (w/wo TPANI additive)‐based mini‐module (5 × 5 cm substrate with effective area of 14.1 cm^2^). (i) *J‐V* curves of the wide bandgap (1.79 eV) devices based on the perovskite w/wo TPANI additive.

For comparison, TPA2P was evaluated against the widely studied carbazole‐based SAM, 4PADCB (Figure S25). The 4PADCB‐modified device achieved a PCE of 25.70%, with a *V_OC_* of 1.171 V, *J_SC_* of 25.69 mA cm^−^^2^, and *FF* of 85.41%, confirming the superior performance of TPA2P across key photovoltaic parameters. Devices fabricated without the TPA2P SAM—whether with or without TPANI—showed significantly lower PCEs (14.09% and 11.72%, respectively), highlighting the critical role of TPA2P in forming an ordered, crystallized heterointerface and confirming its interfacial presence.

Long‐term stability tests under accelerated aging conditions (maximum power point tracking at 40°C ± 5°C under AM 1.5G illumination in N_2_) demonstrated the durability of TPANI‐based devices. Unencapsulated TPANI devices retained 97.2% of their initial PCE after 1000 h, whereas control devices maintained only 91.1% (Figure 4d). This clear stability advantage emphasizes the ability of the crystallized heterointerface to suppress moisture diffusion and mitigate interfacial degradation under continuous illumination.

Electrochemical impedance spectroscopy provided further insight into charge transport and recombination behavior. TPANI‐based devices exhibited a lower series resistance (R_S_  =  0.12 vs. 1.77 Ω cm^2^ for the control) and a higher recombination resistance (R_rec_ =  5675 vs. 2491 Ω cm^2^), along with an extended recombination lifetime (τ_rec_ =  0.398 vs. 0.224 ms) (Table S4 and Figure 4e). These results point to improved carrier transport and suppressed interfacial non‐radiative recombination.

Mott–Schottky analysis further revealed an increased built‐in potential (V_bi _ =  0.99 vs. 0.96 V for the control; Figure 4f), indicating a strengthened internal electric field at the HTL/perovskite interface that enhances charge separation and extraction. Although this increase alone cannot fully explain the observed *V_OC_* enhancement, it is consistent with improved interfacial energetics and reduced non‐radiative losses, corroborating the higher *V_OC_* and *FF* observed experimentally.

Transient photovoltage (TPV) and transient photocurrent (TPC) measurements (Supporting Information Note 5, Figures 4g, and S26) provided additional kinetic evidence for reduced recombination. The TPANI device exhibited a shorter TPC decay time (0.34 vs. 1.30 µs for the control), reflecting faster charge extraction. The corresponding TPV lifetime was also longer (32.24 vs. 19.64 µs), consistent with inhibited non‐radiative recombination.

Light‐intensity‐dependent characteristics further confirmed the suppression of trap‐assisted recombination (Supporting Information Note 6 and Figure S27). The *V_OC_*‐light intensity slope of 1.16 kT/q for the TPANI device was close to the ideal value and significantly lower than that of the control (1.52 kT/q), indicating that trap‐assisted Shockley–Read–Hall (SRH) processes were effectively suppressed. Similarly, the *J_SC–_* light intensity fitting yielded a power‐law exponent (α) of 1.016 for the TPANI device (vs. 0.977 for the control), indicative of minimal bimolecular recombination and efficient charge collection with negligible space‐charge limitations.

To assess scalability, larger‐area devices (1.008 cm^2^, Figure S28) and mini‐modules (5.0 × 5.0 cm substrates, active area  =  14.1 cm^2^, Figures 4h and S29) were fabricated. The large‐area TPANI device achieved a PCE of 25.10%, outperforming the control (21.74%), while the TPANI mini‐module reached a PCE of 20.03%, compared to 18.65% for the control, with improvements in both *V_OC_* and *FF*.

Finally, the general applicability of this interfacial engineering strategy was verified using a wide‐bandgap perovskite (1.79 eV, Cs_0_._2_FA_0_._8_PbI_1.8_Br_1.2_). The TPANI‐modified wide‐bandgap device achieved a PCE of 20.25%, representing a relative enhancement of more than 13% over the control (17.55%; Figure 4i). These results collectively demonstrate that the crystallized heterointerface enabled by TPANI is a versatile and effective design strategy for realizing high‐efficiency, scalable, and stable perovskite photovoltaics.

Based on the complementary TPA‐based molecular pair (TPA2P SAM and TPANI additive) demonstrated in this work, we have established molecular templating as a powerful and generalizable framework for achieving precise structural and energetic matching at buried interfaces, thereby enabling high‐efficiency and durable perovskite photovoltaic devices. To extend this concept to other SAM/additive systems, we propose that at least the following design rules should be satisfied: (i) The SAM and the additive should share a common π‐conjugated core (e.g., triphenylamine, carbazole, or fluorene) to enable effective π–π stacking and non‐covalent electronic coupling at the buried interface; (ii) The SAM must bear a robust anchoring group (e.g., bisphosphonic acid) for strong attachment to the ITO substrate, while the additive should contain a terminal ammonium or similar group to interact with the perovskite surface and passivate defects.

## 3 Conclusion

We demonstrated a non‑covalent molecular templating strategy to engineer the buried interface in inverted PSCs by coupling two complementary triphenylamine‐based molecules: the bisphosphonic acid SAM TPA2P and the ammonium salt additive TPANI. During perovskite crystallization, TPANI migrates to the buried interface, where non‐covalent interactions with TPA2P strengthen adhesion, reduce interfacial roughness, and promote conformal grain growth. This molecularly templated interface minimizes defect density, optimizes band alignment, and enhances charge transport across the SAM/perovskite boundary. Consequently, devices achieve a PCE of 26.58% with a fill factor of 86.72% and retain 97.2% of initial efficiency after 1000 h of operation. These improvements arise from suppressed non‐radiative recombination and improved interfacial stability. This work establishes molecular templating as a powerful, generalizable framework for achieving precise structural and energetic matching at buried interfaces, enabling high‐efficiency and durable perovskite photovoltaic devices.

## Author Contributions

**Songyang Yuan**: investigation, writing – original draft, methodology, software, data curation. **Quanrun Qiu**: methodology, validation. **Huaiman Cao**: methodology, investigation. **Chengda Ge**: investigation, validation. **Yiting Jiang**: data curation. **Gengyang Su**: validation, data curation. **Lingyi Ke**: software, data curation. **Gengxin Du**: data curation. **Guangruixing Zou**: data curation. **Hui Liu**: data curation. **Guanhua Ren**: data curation. **Jing Wang**: data curation. **Nan Zhang**: data curation. **Yidan An**: data curation. **Qingduan Li**: resources. **Ze Yu**: methodology, resources. **Tao Jia**: methodology, resources. **Yue‐Peng Cai**: resources. **Shengjian Liu**: funding acquisition, resources. **Hin‐Lap Yip**: conceptualization, funding acquisition, resources, writing – review and editing, supervision, project administration, methodology.

## Conflicts of Interest

The authors declare no conflicts of interest.

## Supporting information



(Materials and methods; experimental details; additional simulation details and characterization data, including XRD, SEM, Tensile testing, XPS, binding energy and charge density difference calculation by DFT method, AFM, UPS, TDOS, PL mapping, *J‐V*, SPO, TPV, light intensity dependence of *V*_OC_, and photograph of large area device.)**Supporting File**: anie74075‐sup‐0001‐SuppMat.docx.

## Data Availability

The data that support the findings of this study are available from the corresponding author upon reasonable request.
