# Supplementary material for: Molecularly Templated Buried Interfaces for Inverted Perovskite Solar Cells
Source: Angew Chem Int Ed Engl. 2026 Aug 7;65(41):e6932848. doi: 10.1002/anie.6932848 (PMC13638124; doi:10.1002/anie.6932848)
Supplement: Supplementary file 1 [file ANIE-65-e6932848-s001.docx]

**Supporting Information**

**Molecularly Templated Buried Interfaces for Inverted Perovskite Solar Cells**

*Songyang Yuan^a,b,‡^, Quanrun Qiu^b,‡^, Huaiman Cao^b,c,‡^, Chengda Ge^b^, Yiting Jiang^b^, Gengyang Su^d^, Lingyi Ke^b^, Gengxin Du^b^, Guangruixing Zou^b^, Hui Liu^b^, Guanhua Ren^b^, Jing Wang^b^, Nan Zhang^b^, Yidan An^b^, Qingduan Li^a^,* *Ze Yu^c,*^, Tao Jia^a,*^, Yue-Peng Cai ^a^, Shengjian Liu^a,*^ and Hin-Lap Yip^b,e,f,g,*^*

^a^School of Chemistry, Guangzhou Key Laboratory of Materials for Energy Conversion and Storage, Key Laboratory of Electronic Chemicals for Integrated Circuit Packaging, South China Normal University (SCNU), Guangzhou 510006, China

^b^Department of Materials Science and Engineering, City University of Hong Kong, Kowloon, Hong Kong 999077, China

^c^State Key Laboratory of Fine Chemicals, Frontiers Science Center for Smart Materials Oriented Chemical Engineering, School of Chemical Engineering, Dalian University of Technology, Dalian 116024, P. R. China

^d^Guangdong Zenithnano New material co., Ltd. Guangzhou 510006, China

^e^Hong Kong Institute for Clean Energy (HKICE), City University of Hong Kong, Kowloon, Hong Kong 999077, China

^f^State Key Laboratory of Marine Environmental Health, City University of Hong Kong, Kowloon, Hong Kong 999077, China

^g^School of Energy and Environment, City University of Hong Kong, Kowloon, Hong Kong 999077, China

^*^Corresponding authors. Email: [ze.yu@dlut.edu.cn](mailto:ze.yu@dlut.edu.cn) (Ze Yu); tjia@gpnu.edu.cn (Tao Jia); shengjian.liu@m.scnu.edu.cn (Shengjian Liu); a.yip@cityu.edu.hk (Hin-Lap Yip);

^‡^These authors contributed equally to this work.

**1. Materials and Methods**

**Materials:**

Metal halide precursors including cesium iodide (CsI), methylammonium iodide (MAI), methylammonium chloride (MACl), lead (II) chloride (PbCl₂), lead bromide (PbBr_2_, 99.9%), 2-Phenylethylamine Hydroiodide (PEAI, 99.99%), C_60_ (99.99%) and 2,9-Dimethyl-4,7-diphenyl-1,10-phenanthroline (BCP, 99.99%) were obtained from Xi'an Polymer Light Technology Corp. High-purity lead iodide (PbI₂, 99.9985%), and 4PADCB were supplied by Tokyo Chemical Industry Co., Ltd. (TCI). Formamidinium iodide (FAI) was sourced from Dysol Co., Ltd. N,N-dimethylformamide (DMF, 99.99%), dimethyl sulfoxide (DMSO, 99.70%), isopropanol (IPA, 99.50%), and chlorobenzene (CB, 99.90%) were procured from J&K Scientific. All chemical reagents were employed as received without further purification.

**Perovskite Precursor Preparation**

*Normal Bandgap:*

The formula of normal bandgap precursor solution refers to the previous published work.^[^^1^^]^ A 1.4 M perovskite precursor solution with a nominal composition Cs₀.₀₅FA₀.₉₅PbI₃ was prepared by dissolving stoichiometric amounts of FAI, PbI₂, and CsI in a mixed solvent of DMF and DMSO (volume ratio 4:1). The precursor solution was supplemented with excess PbI₂ (5 wt%), PbCl₂ (3 wt%), and MACl, (10 wt%). The resulting solution was stirred at room temperature for at least 1 hour with no subsequent filtration before use. For the doped solution, around 0.25 – 10 mg TPANI was added to this solution.

*1.79 eV Wide-Bandgap (WBG):*

The 1.79 eV WBG perovskite precursor solution was prepared following a similar procedure, CsI (62.4mg) FAI (165.1 mg) PbI₂ (221.3 mg), PbBr₂ (264.2 mg), MACl (1.62 mg) and PbCl_2_ (6.67 mg) dissolved in 1 mL of DMF/DMSO mixed solvent (3:1 v/v). The solution was stirred overnight at room temperature, and no filtration was required before use.

**Device Fabrication**

*Single-junction normal bandgap PSCs:*

The pre-patterned ITO glass substrates were subjected to a rigorous cleaning process involving sequential sonication with detergent, deionized water, acetone, IPA, each for 15 minutes. Following this, the cleaned ITO substrates were dried in an oven at 75 °C for 24 hours and treated with oxygen plasma for 30 minutes prior to use. The solution of SAM materials (TPA2P) was prepared by dissolving with IPA to a concentration of 0.75 mg/mL, respectively. This solution was spin-coated onto the ITO substrates at 4,000 rpm for 30 seconds, followed by annealing at 100 °C for 10 minutes. Subsequently, 45 μL of the perovskite precursor was spin-coated at 500 rpm for 5 s 4,000 rpm for 30 seconds, with 150 μL of CB antisolvent was introduced at the film 15 seconds before the end of the spin-coating process. This was followed by annealing at 100 °C for 50 minutes. Then, 70 μL of PDADI solution (1 mg/mL in isopropanol) was spin-coated onto the perovskite surface at 5000 rpm for 30 s and then annealed at 100 °C for 10 min. All spin-coating processes were conducted in a nitrogen-filled glovebox, maintaining O_2_ and H_2_O levels below 5 ppm, at a controlled temperature of approximately 20 °C. Finally, 25 nm layer of C_60_, 6 nm layer of BCP, and 100 nm Ag were thermally evaporated in a high-vacuum chamber (< 2 × 10⁻⁶ torr) through a metal shadow mask with an aperture area of 0.04 cm². This was followed by the thermal evaporation of a 100 nm MgF_2_ layer onto the glass side of the devices, serving as an antireflection layer.

*Single-junction 1.79 eV bandgap PSCs:*

The perovskite layers were prepared using a two-step spin-coating process with 1000 rpm for 10 s and a second step 4000 rpm for 40 s. During the spin-coating, 180 μL of chlorobenzene (CB) antisolvent was carefully dripped onto the wetted film 20 s before the process concluded. The film was then annealed at 100 °C for 10 minutes. Subsequently, a passivation layer was applied by spin-coating PEAI solution (0.5 mg/mL in isopropanol) at 3000 rpm for 30 s, followed by annealing at 100 °C for an additional 10 minutes.

*Large area (1.008 cm^2^) normal bandgap PSCs:*

Patterned ITO substrates were treated with oxygen plasma for 30 minutes and immediately transferred into an N₂-filled glovebox. A solution of the self-assembled monolayer (SAM) was evenly applied onto the ITO substrates, followed by spin-coating at 3000 rpm for 30 s and subsequent annealing at 100 °C for 10 min to form the hole transport layer. After cooling, 80 μL of the perovskite precursor solution was spin-coated on the SAM/ITO substrate at 1000 rpm for 10 s and then at 4000 rpm for 60 s. Chlorobenzene (300 μL) was dripped onto the center of the substrate 5 seconds before the end of the spin-coating process. The resulting perovskite films were immediately transferred to a hotplate and annealed at 100 °C for 30 min. For the 2D passivation layer, 70 μL of a PDADI solution (1 mg/mL in isopropanol) was spin-coated onto the perovskite surface at 5000 rpm for 30 s and then annealed at 100 °C for 10 min. Finally, layers of C₆₀ (25 nm), BCP (6 nm), and Ag (100 nm) were thermally evaporated through a shadow mask under high vacuum (< 2 × 10⁻⁶ Torr).

*Mini-module normal bandgap PSCs fabrication:*

Perovskite solar films were fabricated by spin coating inside a N_2_ glovebox. The ITO substrates (5*5 cm^2^) with P1 line etching were cleaned like those for small area devices. The substrates were treated with oxygen plasma for 30 min and transferred into an N_2_-filled glove box immediately, The SAM solution was dripped evenly on ITO substrates and spin-coated at 3000 rpm for 30 s and then annealed at 100 °C for 10 min to achieve the deposition of the holt transport layer. After cooling to room temperature, 210 μL of the prepared perovskite precursor solution was dripped on the SAM-coated substrate and spread evenly by the pipette tip, then spun coated at 1000 rpm for 10 s and 4000 rpm for 60 s. CB (400 μL) was added dropwise onto the center of substrates at 4 s before the end of spin coating program. The as-prepared perovskite films were immediately transferred to a 100 °C hotplate and annealed for 30 min. After cooling, 120 μL of PDADI solution (1 mg/mL in isopropanol) was spin-coated onto the perovskite surface at 5000 rpm for 30 s with subsequent annealing treatment at 100 °C for 10 min. Finally, a 25 nm C_60_, 6 nm BCP and 100 nm Ag were thermally evaporated in a high-vacuum chamber (< 2 × 10^-6^ torr). Two laser-scribing processes were performed after thermal evaporation by using a laser-marking machine (532 nm). The widths of the laser-scribed lines for P2 (2.5 W, 100 kHz) and P3 (5 W, 100 kHz) were 70 μm and 50 μm, respectively.

**Characterizations**

*Film characterizations:*

Tensile testing was conducted with a Tensile Testing Machine (DR-5010AQ) from Dongguan dongriyiqi Corp. Ultraviolet photo-electron spectroscopy (UPS) and X-ray photoelectron spectroscopy (XPS) analysis was conducted using a Thermo Fisher ESCALAB XI+ spectrometer, utilizing non-monochromatic He I UV light with an energy of 21.21 eV acting as the excitation source at an energy resolution of 50 meV. Powder and thin-film X-ray diffraction (XRD) characterizations were conducted using a D2 Phaser instrument with Cu Kα radiation (wavelength of 1.5418 Å). Atomic force microscope (AFM) and Kelvin probe force microscopy (KPFM) data were acquired via Bruker Dimension Kelvin probe force microscopy in Potential Channel equipped with PFQNE-AL probe. Fourier-transform infrared (FTIR) spectroscopy was conducted by Fourier transform infrared spectrometer (Tensor 27, Germany Bruker). FTIR spectra were taken from KBr pellet-pressed power samples to amplify the signal. The contact angle measurements were performed using a drop shape analyzer (DSA 25S, Kruss). PL mapping was acquired using a laser scanning confocal Raman spectrometer (LabRAM HR Evolution) with excitation at 405 nm on the perovskite thin film. Steady-state photoluminescence (PL), time-resolved PL (TRPL), and photoluminescence quantum yield (PLQY) spectra were measured using an FLS1000 photoluminescence spectrometer system (Edinburgh). Excitation was achieved with 375 nm light for PL and a pulsed 375 nm laser for TRPL. The dark J–V characteristics of the hole only devices were measured using a Keithley 2400 SourceMeter.

*Device characterizations:*

The current density-voltage (J-V) characteristics of the devices were measured in a nitrogen-filled glovebox using Keithley 2400 Source Meter, under simulated sunlight (SS-F5, EnliTech). To achieve an AM 1.5G (100 mW/cm²) solar simulator light intensity, a National Renewable Energy Laboratory (NREL) calibrated silicon solar cell with a KG-2 filter was used. During the J-V testing, perovskite solar cells were covered with a shading mask with an aperture area of 0.04 cm² to ensure the accuracy of the current density derived from the J-V curves. The J-V measurements were performed in sweep mode with both reverse and forward scans at a scan rate of 10 mV/s and a step size of 0.02 V. Additionally, external quantum efficiency (EQE) curves were obtained using an EQE measurement system (QE-R, EnliTech). The PSCs were operated at their maximum power point (MPP) while being illuminated by an LED source covering wavelengths from 400 to 1,000 nm. Throughout the test, the sample chamber was maintained in a nitrogen-filled glovebox. Electrical impedance spectroscopy (EIS) measurement was carried out using electrochemical analyzer CHI760E (Chenhua Co. Ltd, Shanghai, China) at room temperature. The TPV technique using PAIOS was based on monitoring the photovoltage decay upon a small optical perturbation during different constant bias light-intensity. Transient photovoltage (TPV) and transient photocurrent (TPC) measurement was performed with a LED light illumination and a small perturbation 500 nm laser pulse (pulse width of ~7 ns). The laser is supplied by a commercial Nd:YAG OPO (Oppolette), at a constant repetition rate of 20 Hz. The photocurrent is generated with a photovoltage signal coupled to a 50 Ω resistor, recorded by an oscilloscope (Tektronix TDS 3052C).

**2. Supplementary Notes**

**Supplementary Note 1. First-principles calculations.**

The electron density distributions are calculated by density functional theory (DFT) method with the software of CP2K v2024.1 program utilizing a DZVP-MOLOPT-SR-GTH basis and the PBE exchange-correlation functional.^[^^2-3^^]^ The energy cut-off for the electron density expansion is 350 Ry, and the convergence threshold of density matrix of inner self-consistent field (SCF) is 10^-6^ eV and 0.05 eV Å^−1^ for the norm of the atomic forces.

*Density of states analysis:*

The total density of states (TDOS) and projected density of states (PDOS) are performed within the framework of DFT calculation, with wavefunction data extracted from SCF converged charge densities. The date post-processing and visualizing were used the Multiwfn 3.8 and VMD (Visual Molecular Dynamics) 1.9.4 package, respectively.^[^^4-6^^]^

*Highest occupied molecular orbital (HOMO) and Lowest unoccupied molecular orbital (LUMO) analysis:*

The HOMO and LUMO of single SAM materials were calculated using ORCA 5.0.1. software.^[^^7^^]^ The geometry optimization at the level of theory of B3LYP/def2-SVP. Then, total energies were further calculated at the level of theory of B3LYP/def2-TZVP based on single-point calculations of the optimized structures.

*The Binding energy calculation:*

For the binding energy calculation, the binding energy (${\Delta E}_{binding}$) is defined as:

${\Delta E}_{binding}=E_{slab+molecule}-E_{slab}-E_{molecule}$ (1)

Here, $E_{slab}$ and $E_{molecule}$ are the total energy of the slab and adsorbed SAM molecule, respectively.

*The charge density differential analysis:*

The charge density differential analysis is performed to elucidate the charge redistribution characteristics during the interfacial interaction. The charge density differential (Δρ) is calculated using the formula:

$\Delta\rho=\rho_{slab+molecule}-\rho_{slab}-\rho_{molecule}$ (2)

where $\rho_{slab+molecule}$ represents the electron density of the optimized system, $\rho_{slab}$ and $\rho_{molecule}$ are the electron density of PVK substrate and adsorbed molecule in the optimized system. To ensure computational consistency, all atomic positions in the isolated state maintained identical coordinates to those in the optimized configuration. The charge density distributions are visualized through isosurface analysis with a threshold value of 0.02 e/Å³.

**Supplementary Note 2: Time-resolved photoluminescence (TRPL) analysis.**

The passivation effect on the perovskite film was evaluated using TRPL spectroscopy. The TRPL decay curves were fitted with a biexponential function of the form:^[^^8-9^^]^

$y=y_{0}+A_{1}e^{(\frac{-\tau}{\tau_{1}})}+A_{2}e^{(\frac{-\tau}{\tau_{2}})}$ (3)

Where *A_1_* and *A_2_* represent the amplitudes of the carrier lifetime components *τ_1_* and *τ_2_*, respectively. *y*_0_ is a constant offset, The time decay components *τ*_1_ and *τ*_2_ associated with fast bimolecular recombination, while *τ*_2_ corresponds to slower trap-assisted recombination. The average carrier recombination lifetime (*τ_avg_*) can be calculated by equation:

$\tau_{avg}=\frac{\sum A_{i}\tau_{i}^{2}}{\sum A_{i}\tau_{i}}$ (4)

A larger *τ_avg_* could be index for better quality of perovskite due to the suppression of defect states.

**Supplementary Note 3: Calculation of the quasi-Fermi level splitting (QFLS) based on the PL quantum yield (PLQY).**

To evaluate the influence of TPANI additive on non-radiative recombination in the perovskite precursor solution, the QFLS of 1.53 eV bandgap perovskite films on ITO/TPA2P substrates was determined through PLQY measurements. The relationship between QFLS and PLQY is given by:^[^^10^^]^

$QFLS={QFLS}_{rad}+K_{B}Tln\left( PLQY \right)=K_{B}\mathrm{Tln}\left( \frac{J_{G}}{J_{0,rad}}PLQY \right)$ (5)

Here, *QFLS* is the difference between the electron and hole quasi-Fermi levels in perovskite layer, *k_B_* is the Boltzmann constant, and *T* is the Kelvin temperature (300 K). *J_G_* is the generation current density under illumination. In this case, it is approximated to the short-circuit current density *J_SC_* of the devices. *J_0,rad_* is the dark radiative recombination saturation current density.

According to the detailed balance theory, the *J*_0_*_,rad_* could be calculated by the following equations:

$J_{0,rad}=q\int_{0}^{\infty} {EQE}_{PV}\left( E \right)\phi_{BB}\left( E \right)dE$ (6)

$\phi_{BB}\left( E \right)=\frac{{2\pi E}^{2}}{h^{3}c^{2}}*\frac{1}{\exp\left( \frac{E}{K_{B}T} \right)-1}$ (7)

Where *q* is the elementary charge, *EQE_PV_* is the photovoltaic external quantum efficiency, *E* is the photon energy, *𝜙_BB_* is the black-body radiative spectrum, *h* is the Planck constant, and *c* is the light speed in vacuum.

External quantum efficiency (EQE) of the PSCs and the emitted spectral photon flux could be calculated when the device is in equilibrium with the black-body radiation at 300 K. Based on equations 6 and 7, the *J_0,rad_* is calculated similarly as 1.72 × 10^-19^ and 1.69 × 10^-19^ A*m^−2^ for the samples that w/wo the TPANI addictive in perovskite precursor solution, respectively. Subsequently, combined with the corresponding PLQY value of the samples, the QFLS could be obtained according to equation 5. Besides, the V_OC_ non-radiation recombination loss ${\Delta V}_{OC}^{non-rad}$ could be obtained according to the following equation:

${\Delta V}_{OC}^{non-rad}=\frac{{QFLS}_{rad}-QFLS}{q}=\frac{k_{B}Tln(PLQY)}{q}$ (8)

**Supplementary Note 4: SCLC analysis.**

The dark J–V characteristics of the hole-only devices can be characterized by three distinct regimes: the Ohmic region, the trap-filling limited (TFL) region, and the trap-free Child region. The trap density (*N_t_*) in perovskite films, with and without the TPANI additive, was estimated using the following equation:^[^^11^^]^

$N_{t}=\frac{2\varepsilon\varepsilon_{0}V_{TFL}}{qd^{2}}$ (9)

where *d* is the thickness of the perovskite film, *q* is the elementary charge (1.602 × 10^-19^ C), *ε* is the relative dielectric constant of the perovskite material, and *ε_0_* is the vacuum permittivity (8.8542 × 10^-14^ F/cm). *V_TEL_* denotes the trap-filling limit voltage. The relative dielectric constant *ε* was determined through impedance spectroscopy using the equation:^[^^12-13^^]^

$\varepsilon=\frac{Cd}{A\varepsilon_{0}}$ (10)

where C is capacitance at high frequency (~10^-4^ Hz), *d* is the thickness of perovskite film, *A* is active area. Furthermore, the charge mobility (*μ*) is extracted by the Mott-Gurney Law:

$\mu=\frac{8J_{D}d^{3}}{9\varepsilon\varepsilon_{0}V^{2}}$ (11)

where *J_D_* is the dark current density and *V* is the applied voltage.

**Supplementary Note 5. TPV and TPC analysis.**

The TPV and TPC could be fitted with mono-exponential decay equation:^[^^14^^]^

$R_{t}=y_{0}+B_{1}e^{(\frac{-\tau}{\tau_{1}})}$ (12)

Where *B_1_* is the correlation coefficient of carrier lifetime *τ_1_*.

**Supplementary Note 6: *J_SC_* and *V_OC_* under different light intensity.**

To further probe the charge carrier recombination mechanisms, the dependence of both *J_SC_* and *V_OC_* on light intensity was examined. The ideality factor (*n*_ID_) was derived from the slope of the *V_OC_* versus light intensity plot:^[^^15^^]^

$slope=\frac{n_{ID}k_{B}T}{q}$ (13)

where *q*, *k_B_*, and *T* denote the elementary charge, Boltzmann's constant, and Kelvin temperature.^[^^16^^]^ the value of *n*_ID_ closing to 1 indicates that the charge carrier recombination in the devices is suppressed.^[^^17^^]^

We also examined the exponent α, which serves as an indicator of the current-density dependence on light intensity. A value of α close to 1 suggests the absence of bimolecular recombination in the device.^[^^18^^]^

**3. Supplementary Figures**

**Figure S1.** Single crystal structures of 2D perovskite (TPA)_2_PbI_4_ viewing from different direction. (a) a side, (b) b side and (c) c side.

**Figure S2.** XRD patterns of perovskite films with different concentration of TPANI additive.

**Figure S3.** (a) XRD patterns of perovskite films with different concentration of TPANI additive. (b) An enlarged view of the red box in Figure a, showing the existence of 2D perovskite (TPA)_2_PbI_4_. The (002) and (003) peak are at 7.1° and 10.5°, respectively.

**Figure S4.** The SEM image of the surface of PVK film with different concentration of TPANI additive.

**Figure S5.** Tensile testing by peeling off perovskite (TPANI) film from ITO/4PADCB substrates.

**Figure S6.** Adhesion energy of perovskite films deposited on different SAM layer w/wo TPANI as additive. Each group consists of 5 pieces to ensure the statistics accuracy.

**Figure S7.** Optical photograph of the pieces after being peeled off perovskite film via the tensile testing method. Each group consists of 5 pieces to ensure the statistics accuracy.

**Figure S8.** The Pb 4f XPS spectra of ITO/TPA2P substrate after being peeled off perovskite film w/wo TPANI additive.

**Figure S9.** (a) XPS depth profile of N 1s core level of buried interface perovskite film with TPANI additive. (b) N 1s core level of surface perovskite film with TPANI additive. perovskite film with TPANI additive. The perovskite film with TPANI additive was deposited on ITO substrate. Etching initiated from the buried interface of perovskite film with 2000 eV etching intensity.

**Figure S10.** (a) Adsorption Configuration of the (a) TPAN^+^ and (b) TPA2P molecules on PVK surface.

**Figure S11.** The binding energy of TPAN^+^ and TPA2P molecules on PVK surface.

**Figure S12.** (a) The 3D visual charge density difference of the (a) TPAN^+^ and (b) TPA2P molecules on PVK surface. Electron depletion/accumulation is depicted by the cyan/yellow isosurfaces. at ±0.002 |e| Å^−3^.

**Figure S13.** (a-d) The 3D AFM images of buried PVK interface w/wo TPANI additive.

**Figure S14.** The AFM images in different area of the buried PVK interface (a-d) without or (e-f) with TPANI additive.

**Figure S15.** The AFM images of the surface of perovskite film w/wo TPANI additive.

**Figure S16.** Cutoff region of UPS spectra for bare lTO, ITO/TPA2P and ITO/TPA2P substrates after being peeled off perovskite film w/wo TPANI additive. a.u., arbitrary units, WF, work function.

**Figure S17.** The edge states of TDOS and PDOS (Pb-6s and C-2p) distribution for PVK substrate and PVK substrate adsorbing one TPAN^+^ ion.

**Figure S18.** 3D PL mapping of top view perovskite films w/wo TPANI additive.

**Figure S19.** (a) PLQY and QFLS of perovskite films w/wo TPANI additive.

**Figure S20.** Statistics of device performance parameters for PSCs with different concentrations of TPANI additive in PVK precursor solution ranging from 0 mg/ml to 1.0 mg/ml.

**Figure S21.** Statistics of device performance parameters for PSCs with high concentrations of TPANI additive in PVK precursor solution ranging from 0 mg/ml to 10 mg/ml.

**Figure S22.** Stabilized power output characteristics of the device w/wo TPANI additive in PVK precursor solution.

**Figure S23.** d(EQE)/dE versus wavelength for PVK (TPANI) based devices.

**Figure S24.** *J-V* curves of the perovskite (w/wo TPANI additive)-based devices at forward scan.

**Figure S25.** Statistics of device performance parameters for PSCs with different SAM layers (or SAM layer free).

**Figure S26.** TPV carrier recombination lifetimes for the device w/wo TPANI additive in PVK precursor solution.

**Figure S27.** Light intensity-dependent measurements of (a) *V_OC_* and (b) *J_SC_* for the device w/wo TPANI additive in PVK precursor solution.

**Figure S28.** (a) Photograph and (b) PCE performance of large area PSCs (1.008 cm^2^).

**Figure S29.** Photograph of mini-module (5 cm * 5 cm substrate with effective area of 14.1 cm^2^).

**Table S1.** TRPL fit parameters of the perovskite films with or without TPANI on a structure of ITO/TPA2P/perovskite. The laser incidents from the perovskite side.

|  | A1 | τ1 (ns) | A2 | τ2 (ns) | τ(average) (ns) |
| --- | --- | --- | --- | --- | --- |
| PVK | 500.41 | 133.83 | 1065.98 | 18.31 | 107.75 |
| PVK (TPANI) | 973.36 | 16.07 | 454.87 | 189.87 | 163.22 |

**Table S2.** Parameters summary of PLQY, QFLS and *V_OC_* loss on Glass an ITO/TPA2P substrate.

| Sample | PLQY (%) | QFLS (eV) | Voc loss (mV) |
| --- | --- | --- | --- |
| ITO/TPA2P/PVK (TPANI) | 8.2 | 1.198 | 64.7 |
| ITO/TPA2P/PVK | 3.8 | 1.179 | 84.5 |
| Glass/PVK (TPANI) | 9.4 | 1.202 | 61.1 |
| Glass/PVK | 7.8 | 1.197 | 65.9 |

**Table S3.** Parameters for the devices w/wo TPANI additive in PVK precursor solution.

| Name | V_max_ (V) | J_max_ (mA/cm^2^) | Rs (Ω) | Rsh (Ω) |
| --- | --- | --- | --- | --- |
| PVK (TPANI) | 1.06 | 24.93 | 53.88 | 142349.7 |
| PVK | 1.04 | 24.68 | 47.16 | 142430.7 |

**Table S4.** Parameters extracted from the EIS data. R_s_, C_rec_, R_rec_, and τ_rec_ stand for series resistance, recombination capacitance, recombination resistance, and recombination lifetime.

|  | R_s_ (Ω/cm^2^) | C_rec_ (F/cm^2^) | R_rec_ (Ω/cm^2^) | τ_rec_ (ms) |
| --- | --- | --- | --- | --- |
| PVK | 1.768 | 8.99E-08 | 2491 | 0.224 |
| PVK (TPANI) | 0.12 | 7.02E-08 | 5675 | 0.398 |

**REFERENCES**

[1]	X. Huang; L. Bi; Z. Yao; Q. Fu; B. Fan; S. Wu; Z. Su; Q. Feng; J. Wang; Y. Hong; M. Liu; Y. An; M. Chen; A. K.-Y. Jen, *Adv. Mater.* **2024**, 2410564.

[2]	T. D. Kühne; M. Iannuzzi; M. Del Ben; V. V. Rybkin; P. Seewald; F. Stein; T. Laino; R. Z. Khaliullin; O. Schütt; F. Schiffmann; D. Golze; J. Wilhelm; S. Chulkov; M. H. Bani-Hashemian; V. Weber; U. Borštnik; M. Taillefumier; A. S. Jakobovits; A. Lazzaro; H. Pabst; T. Müller; R. Schade; M. Guidon; S. Andermatt; N. Holmberg; G. K. Schenter; A. Hehn; A. Bussy; F. Belleflamme; G. Tabacchi; A. Glöß; M. Lass; I. Bethune; C. J. Mundy; C. Plessl; M. Watkins; J. VandeVondele; M. Krack; J. Hutter, *J. Chem. Phys.* **2020**, *152*, 194103.

[3]	M. J. van Setten; M. Giantomassi; E. Bousquet; M. J. Verstraete; D. R. Hamann; X. Gonze; G. M. Rignanese, *Comput. Phys. Commun.* **2018**, *226*, 39-54.

[4]	T. Lu, *J. Chem. Phys.* **2024**, *161*, 082503.

[5]	T. Lu; F. Chen, *J. Comput. Chem.* **2012**, *33*, 580-592.

[6]	W. Humphrey; A. Dalke; K. Schulten, *J. Mol. Graphics* **1996**, *14*, 33-38.

[7]	F. Neese, *WIRES COMPUT MOL SCI* **2022**, *12*, e1606.

[8]	Q. Hu; E. Rezaee; W. Xu; R. Ramachandran; Q. Chen; H. Xu; T. EL-Assaad; D. V. McGrath; Z.-X. Xu, *Small* **2021**, *17*, 2005216.

[9]	G. Qu; D. Khan; F. Yan; A. Atsay; H. Xiao; Q. Chen; H. Xu; I. Nar; Z.-X. Xu, *J. Energy Chem.* **2022**, *67*, 263-275.

[10]	M. Stolterfoht; P. Caprioglio; C. M. Wolff; J. A. Márquez; J. Nordmann; S. Zhang; D. Rothhardt; U. Hörmann; Y. Amir; A. Redinger; L. Kegelmann; F. Zu; S. Albrecht; N. Koch; T. Kirchartz; M. Saliba; T. Unold; D. Neher, *Energ. Environ. Sci.* **2019**, *12*, 2778-2788.

[11]	W. Zhou; S. Tai; Y. Li; H. Fu; Q. Zheng, *Adv. Funct. Mater.* **2024**, *34*, 2407897.

[12]	A. A. Zhumekenov; M. I. Saidaminov; M. A. Haque; E. Alarousu; S. P. Sarmah; B. Murali; I. Dursun; X.-H. Miao; A. L. Abdelhady; T. Wu; O. F. Mohammed; O. M. Bakr, *ACS. Energy. Lett.* **2016**, *1*, 32-37.

[13]	V. V. Brus; A. K. K. Kyaw; P. D. Maryanchuk; J. Zhang, *Prog. Photovolt: Res. Appl.* **2015**, *23*, 1526-1535.

[14]	P. Zhu; D. Wang; Y. Zhang; Z. Liang; J. Li; J. Zeng; J. Zhang; Y. Xu; S. Wu; Z. Liu; X. Zhou; B. Hu; F. He; L. Zhang; X. Pan; X. Wang; N.-G. Park; B. Xu, *Science* **2024**, *383*, 524-531.

[15]	J. Liu; J. Chen; L. Xie; S. Yang; Y. Meng; M. Li; C. Xiao; J. Zhu; H. Do; J. Zhang; M. Yang; Z. Ge, *Angew. Chem. Int. Ed.* **2024**, *63*, e202403610.

[16]	I. Riedel; J. Parisi; V. Dyakonov; L. Lutsen; D. Vanderzande; J. C. Hummelen, *Adv. Funct. Mater.* **2004**, *14*, 38-44.

[17]	Q. Cao; Y. Li; H. Zhang; J. Yang; J. Han; T. Xu; S. Wang; Z. Wang; B. Gao; J. Zhao; X. Li; X. Ma; S. M. Zakeeruddin; W. E. I. Sha; X. Li; M. Grätzel, *Sci. Adv.* **2021**, *7*, eabg0633.

[18]	G. Qu; L. Dong; Y. Qiao; D. Khan; Q. Chen; P. Xie; X. Yu; X. Liu; Y. Wang; J. Chen; X. Chen; Z.-X. Xu, *Adv. Funct. Mater.* **2022**, *32*, 2206585.
